# Incidence and Variation of Discrepancies in Recording Chronic Conditions in Australian Hospital Administrative Data

**DOI:** 10.1371/journal.pone.0147087

**Published:** 2016-01-25

**Authors:** Hassan Assareh, Helen M. Achat, Joanne M. Stubbs, Veth M. Guevarra, Kim Hill

**Affiliations:** 1 Epidemiology and Health Analytics, Western Sydney Local Health District, Sydney, Australia; 2 Executive Medical Services, Western Sydney Local Health District, Sydney, Australia; Azienda Ospedaliero-Universitaria Careggi, ITALY

## Abstract

Diagnostic data routinely collected for hospital admitted patients and used for case-mix adjustment in care provider comparisons and reimbursement are prone to biases. We aim to measure discrepancies, variations and associated factors in recorded chronic morbidities for hospital admitted patients in New South Wales (NSW), Australia. Of all admissions between July 2010 and June 2014 in all NSW public and private acute hospitals, admissions with over 24 hours stay and one or more of the chronic conditions of diabetes, smoking, hepatitis, HIV, and hypertension were included. The incidence of a non-recorded chronic condition in an admission occurring after the first admission with a recorded chronic condition (index admission) was considered as a discrepancy. Poisson models were employed to (i) derive adjusted discrepancy incidence rates (IR) and rate ratios (IRR) accounting for patient, admission, comorbidity and hospital characteristics and (ii) quantify variation in rates among hospitals. The discrepancy incidence rate was highest for hypertension (51% of 262,664 admissions), followed by hepatitis (37% of 12,107), smoking (33% of 548,965), HIV (27% of 1500) and diabetes (19% of 228,687). Adjusted rates for all conditions declined over the four-year period; with the sharpest drop of over 80% for diabetes (47.7% in 2010 vs. 7.3% in 2014), and 20% to 55% for the other conditions. Discrepancies were more common in private hospitals and smaller public hospitals. Inter-hospital differences were responsible for 1% (HIV) to 9.4% (smoking) of variation in adjusted discrepancy incidences, with an increasing trend for diabetes and HIV. Chronic conditions are recorded inconsistently in hospital administrative datasets, and hospitals contribute to the discrepancies. Adjustment for patterns and stratification in risk adjustments; and furthermore longitudinal accumulation of clinical data at patient level, refinement of clinical coding systems and standardisation of comorbidity recording across hospitals would enhance accuracy of datasets and validity of case-mix adjustment.

## Introduction

Routinely collected data for hospital admitted patients are increasingly used for clinical and epidemiological research, health resource distribution, funding strategies and quality improvement purposes. Demographic and diagnostic information captured in administrative hospital data collections is employed for case-mix or risk adjustment in order to account for differences in patient characteristics and provide fair comparisons and reimbursements [1–4]. According to certain coding rules and data standards this information is recorded by clinical coders based on patients’ medical information documented during admission [5]. Despite advancements in diagnoses classifications, coding training and standardisation of clinical documentation and coding practices that improved accuracy and reliability of comorbidity information [3, 6], discrepancies in recorded comorbidities at coder, hospital [7–9] and regional levels [10, 11] have been reported in Australia and elsewhere. Relating case mix to funding strategies introduced a systematic bias of reporting more comorbidities, known as “upcoding”, for greater gains in several national health systems [12]. Such biases can change the relationship between patient profile and outcome across hospitals and would potentially lead to inaccurate or unfair provider comparisons and allocation of incentives [2, 4, 13–16].

Different sources of information, employed by studies to verify consistency in hospital datasets, resulted in varying levels of agreement. Higher agreements were reported where hospital data were compared against clinical charts as opposed to self-reported data [7, 17–19]. A recent study reported almost a fifth of the variation in discrepancies in coding common comorbidities in Australian hospitals was attributable to hospital characteristics [7]. Individual hospitals contributed to the observed differences along hospital structural characteristics such as size and location [7, 8, 20].

Despite the important findings from Australian studies previously conducted, no study examined internal consistency of hospital datasets through longitudinal investigation of patient-specific morbidity information. Such a design allows a population-based investigation and reflects discrepancies within a homogeneous setting governed by a single documentation and set of clinical coding standards. Furthermore, investigation of the temporal behaviour of discrepancies and their variations can provide additional insight into the consequences of systematic changes in clinical coding practices such as changes in documentation, coding rules and standards, infra-structure and staffing [8, 21, 22].

This study aimed to measure non-recorded morbidity incidents in administrative hospital datasets and the contribution of patient, admission, morbidity and hospital related factors, as well as examine inter-hospital variation in the observed incidents. We used record linked data for all admitted patients between July 2010 and June 2014 in all acute hospitals across New South Wales (NSW), Australia. Discrepancies in the four chronic conditions of diabetes, hepatitis, HIV and hypertension as well as smoking status were investigated. These five are among most frequently captured conditions in risk-adjustment models [23–25]. Their effect on care and treatment make their recording required or more likely [5].

## Methods

### 2.1 Data source and study population

NSW, the largest health jurisdiction in Australia, has over seven million residents and approximately 500 healthcare facilities with up to three million admissions per annum. We used records from the record linked Admitted Patient Data Collection (APDC) database between 2010–2013 financial years (2010–2013 FY) comprising all NSW hospital separations from 1st July 2010 to 30th June 2014. Each separation (episode of care) record includes information on patient demographics, morbidities and procedures, hospital characteristics, and separations (discharges, transfers and deaths) from all public and private healthcare facilities in NSW. Record linked APDC includes a unique patient identifier that enables the identification and linkage of patient-specific admissions [26]. Each record is assigned with up to 55 codes for morbidities (principal diagnosis and comorbidities) based on the International Statistical Classification of Diseases and Related Health Problems, Tenth Revision, Australian Modification (ICD-10-AM) Seventh Edition [27]. Linked APDC records were obtained from the NSW Admitted Patient, Emergency Department and Deaths Register, which was established under the public health and diseases registers provisions of the NSW Public Health Act 2010 and is maintained by the NSW Ministry of Health. Record linkage was carried out by The Centre for Health Record Linkage (CHeReL)[26]. The data were accessed remotely through Secure Analytics for Population Health Research and Intelligence (SAPHaRI) system made available by Centre for Epidemiology and Evidence, NSW Ministry of Health [28]. De-identified patients’ records were provided and accessed via SAPHaRI and used for analysis. The study was approved by the Western Sydney Local health District (WSLHD) ethics committee and the Centre for Epidemiology and Evidence, NSW Ministry of Health as the data provider.

Of all admissions at all NSW healthcare facilities within our study period (11,278,591 admissions for 3,761,932 patients), we included admissions of those patients who had at least two admissions with hospital length of stays of at least 24 hours in any NSW acute public or private hospital with at least one recorded chronic condition. This study examined 1,545,294 (13.7%) admissions for 385,268 (10.2%) patients. Admissions at community facilities, multipurpose, non-acute or sub-acute centres, psychiatric and rehabilitation facilities, nursing home and hospices, and children’s hospitals were excluded.

### 2.2 Discrepancy identification and covariates

Based on ICD-10-AM, five conditions, diabetes (E10–E14), chronic hepatitis (hepatitis: B18.0-B18.2, B94.2 and Z86.18), chronic HIV (B20-B22, B23.8, and B24), hypertension (I10-I15), and smoking (F17.1, F17.2, Z86.43, and Z72.0), were identified within recorded morbidities for each admission. For each patient, the earliest and the latest admissions with the recorded chronic condition (first and last index admissions respectively) were identified for each chronic condition. A discrepancy incidence in clinical coding was defined as any admission with a non-recorded chronic condition occurring: a) between the first and the last indices; or b) within three months of the last index admission (follow-up period) but not occurring after 31^st^ March 2014 (buffer period). For patients with only one admission with a recorded condition, only one index admission existed and therefore the second criterion was applied. All admissions occurring after the first index admission including the last index admission and those that met the follow-up and buffer periods criteria were included in the denominator.

This restricted prospective approach in the identification of a non-recorded chronic condition was employed to avoid any overestimation caused by counting admissions prior to diagnosis or after possible cure. The limited follow-up period of three months allowed inclusion of any possibly true discrepancy incidents occurring after the last index admission while minimising inclusion of any admission following a false positive admissions (patient had no chronic condition but the condition was recorded). Furthermore a buffer of three months at the end of the study period diminished the effect of censoring among follow-up admissions. An extensive sensitivity analysis using different follow-up periods in the absence or presence of a buffer was conducted and the results were outlined in S1 Table.

For all admissions, four sets of covariates–patient, admission, morbidity, and hospital related–were considered. Patient demographic variables included age, gender and socio-economic status. We utilised a Statistical Local Area level disadvantage index of Socio-Economic Indices for Areas (SEIFA) with the lower values indicating more disadvantage [29]. SEIFA scores were categorised into four classes (1^st^ quartile: most disadvantaged to 4^th^ quartile: least disadvantaged areas). Admission covariates included admission type (surgical, medical, and other), admission source (emergency, planned, and other), and length of stay (1–2, 3–5, 5–10, and over 10 days). Morbidity related factors were number of recorded morbidities categorised by quartiles, presence of any other chronic conditions (yes, no), and discrepancy in recording other four chronic conditions (yes, no). Hospital characteristics included hospital type (public vs. private), location (metropolitan vs. rural), and peer groups for public hospitals. Public hospital peer groups comprised “A1”: principal referral group, usually teaching hospitals; “B”: major metropolitan and non-metropolitan; “C1”: district group 1; and “C2”: district group 2. Hospital peer groups contained similar sized hospitals, ranging from those treating more than 25,000 acute case-mix weighted separations per annum in principal referral groups through to treating between 2,000 and 5,000 acute case-mix weighted separations in district group [30].

### 2.3 Statistical analysis

We employed Poisson linear models to evaluate adjusted discrepancy incidence rates (IR) and rate ratios (IRR) for the five chronic conditions separately after including patient, admission, morbidity and hospital-related characteristics. Separate models for public hospital admissions were constructed to derive estimates for the public hospital peer group effect. Morbidity characteristics were also entered into the models one at a time because of multicollinearity. To investigate the temporal behaviour of the discrepancy incidents, financial years were also entered into the models for all admissions as well as in separate models for public and private hospital admissions as indicator variables, with 2010 as the reference year. Adjusted trends were estimated by multiplying incidence rate ratios obtained from the Poisson model and the crude risk at the reference year. The difference between public and private hospitals trends was also assessed using an interaction term between the hospitals type and year variables in the full model.

Inter-hospital variation among public hospitals was evaluated within a multilevel framework, using a Poisson mixed model with a random intercept component at hospital level for each condition. A series of models were constructed to assess the contribution of hospital-related factors in the observed variation of discrepancy incidents following adjustment from a null model to the most comprehensive with all covariates. To express the inter-hospital variation, we employed the variance partition coefficient (VPC) for Poisson multilevel modelling scheme using the exact formulae developed by Stryhn et al. [31]. The VPC on hospital level indicates the influence of the hospitals on discrepancy incidents that cannot be explained by the model parameters. Due to conditionality of VPC in Poisson modelling on covariates values, the median and inter-quartile of the calculated VPC for all existing covariates values were reported. Furthermore the proportional change in inter-hospital variance estimates ($\sigma_{h}^{2}$) of the different models were calculated. This indicates the proportion of total inter-hospital variation that is explained by case-mix factors. To translate inter-hospital variation into risk differences, we used the median incidence rate ratio (MIRR) statistic which is the median of the rate ratios of pair-wise comparisons of admissions with identical characteristics taken from randomly chosen hospitals and calculated as $\text{exp}(0.95\times \sqrt{\sigma_{h}^{2}})$, an extension of the measure developed by Merlo et al [32, 33]. To assess the effect of hospital size, random intercept estimates were stratified by hospital peer group and associated statistics were derived. To quantify trend of inter-hospital variation over the study period, the Poisson mixed models were extended by the inclusion of the year variable as a categorical random slope. We also used pair-wise Pearson correlation to assess the association of hospital recoding performances across the five chronic conditions, based on the hospital-specific random intercepts. Data preparation was conducted in SAS Enterprise Guide V.6.1 [34] through SAPHaRI [28], and analyses were performed in R package V.3.1.2 [35].

## Results

### 3.1 Discrepancy incidence rate and risk factors

Of 228,687 inspected admissions following 76,666 patients with a diabetes related first index admission, 43,008 subsequent admissions had no recorded diabetes code, resulting in a discrepancy incidence rate of 18.8%. There existed more discrepancy incidents related to the four other chronic conditions: 26.7% (in 1,500 admissions) for HIV, 33.2% (in 182,735 admissions) for smoking, 36.6% (in 12,107 admissions) for hepatitis and the highest rate of 51% (in 262,664 admissions) for hypertension (Table 1).

**Table 1:** Admissions, discrepancies and associated incidence rates and rate ratios, stratified by patient and admission characteristics.

Characteristics	Diabetes			Smoking			Hepatitis			HIV			Hypertension		
	N (%)	n (IR%)	IRR (95% CI)	N (%)	n (IR%)	IRR (95% CI)	N (%)	n (IR%)	IRR (95% CI)	N (%)	n (IR%)	IRR (95% CI)	N (%)	n (IR%)	IRR (95% CI)
**Sex**															
Male	124,569	23,065	1	327,917	107,706	1	8,266	3,002	1	1,345	349	1	134,651	68,499	1
	(54.5%)	(18.5%)	-	(59.7%)	(32.8%)	-	(68.3%)	(36.3%)	-	(89.7%)	(25.9%)	-	(51.3%)	(50.9%)	-
Female	104,118	19,943	0.97 **	221,048	75,029	0.98 **	3,841	1,456	0.97	155	52	0.76	128,013	65,572	0.79 **
	(45.5%)	(19.2%)	(0.95–0.99)	(40.3%)	(33.9%)	(0.97–0.99)	(31.7%)	(37.9%)	(0.91–1.03)	(10.3%)	(33.5%)	(0.56–1.04)	(48.7%)	(51.2%)	(0.78–0.80)
**Age**															
17years and under	1,668	98	0.21 **	2,151	888	1.21 **	8	8	1.85	-	-	-	141	89	0.97
	(0.7%)	(5.9%)	(0.17–0.26)	(0.4%)	(41.3%)	(1.13–1.29)	(0.1%)	(100.0%)	(0.92–3.72)	-	-	-	(0.1%)	(63.1%)	(0.78–1.20)
18–34 years	5,799	1,048	1	50,382	16,411	1	1,088	525	1	144	41	1	2,442	1,376	1
	(2.5%)	(18.1%)	-	(9.2%)	(32.6%)	-	(9.0%)	(48.3%)	-	(9.6%)	(28.5%)	-	(0.9%)	(56.3%)	-
35–54 years	24,065	4,919	1.37 **	104,551	30,567	0.94 **	5,902	2,208	0.77 **	845	233	0.98	17,764	9,276	0.94 *
	(10.5%)	(20.4%)	(1.28–1.46)	(19.0%)	(29.2%)	(0.92–0.96)	(48.7%)	(37.4%)	(0.70–0.85)	(56.3%)	(27.6%)	(0.7–1.39)	(6.8%)	(52.2%)	(0.89–0.99)
55–74 years	92,513	17,416	1.46 **	207,136	64,375	1.05 **	4,331	1,448	0.69 **	464	116	0.94	86,626	43,978	0.92 **
	(40.5%)	(18.8%)	(1.37–1.55)	(37.7%)	(31.1%)	(1.03–1.07)	(35.8%)	(33.4%)	(0.62–0.77)	(30.9%)	(25.0%)	(0.65–1.37)	(33.0%)	(50.8%)	(0.87–0.97)
75–89 years	94,251	17,644	1.57 **	166,793	62,657	1.24 **	745	255	0.73 **	47	11	0.96	131,746	67,250	0.92 **
	(41.2%)	(18.7%)	(1.47–1.67)	(30.4%)	(37.6%)	(1.22–1.27)	(6.2%)	(34.2%)	(0.63–0.86)	(3.1%)	(23.4%)	(0.49–1.90)	(50.2%)	(51.0%)	(0.87–0.97)
90 years and over	10,391	1,883	1.69 **	17,952	7,837	1.4 **	33	14	0.93	-	-	-	23,945	12,102	0.9 **
	(4.5%)	(18.1%)	(1.57–1.82)	(3.3%)	(43.7%)	(1.36–1.44)	(0.3%)	(42.4%)	(0.54–1.58)	-	-	-	(9.1%)	(50.5%)	(0.85–0.95)
**Quartiles of SEIFA**															
1^st^ (most disadvantaged)	66,041	12,220	1	142,286	45,872	1	3,543	1,307	1	162	48	1	67,440	33,840	1
	(28.9%)	(18.5%)	-	(25.9%)	(32.2%)	-	(29.3%)	(36.9%)	-	(10.8%)	(29.6%)	-	(25.7%)	(50.2%)	-
2^nd^	65,977	12,229	1.02	171,257	52,940	0.95 **	2,870	946	0.92 *	263	55	0.7	74,670	37,263	0.98 **
	(28.9%)	(18.5%)	(0.99–1.04)	(31.2%)	(30.9%)	(0.94–0.96)	(23.7%)	(33.0%)	(0.84–1.00)	(17.5%)	(20.9%)	(0.47–1.04)	(28.4%)	(49.9%)	(0.96–0.99)
3^rd^	48,173	8,628	0.98	112,110	36,474	0.98 *	2,895	1,053	0.97	503	144	1.02	54,613	27,521	0.99
	(21.1%)	(17.9%)	(0.95–1.01)	(20.4%)	(32.5%)	(0.97–1.00)	(23.9%)	(36.4%)	(0.89–1.06)	(33.5%)	(28.6%)	(0.72–1.44)	(20.8%)	(50.4%)	(0.97–1.00)
4^th^ (least disadvantaged)	48,496	9,931	1.04 *	123,312	47,449	1.1 **	2,799	1,152	1.03	572	154	0.96	65,941	35,447	0.99
	(21.2%)	(20.5%)	(1.01–1.07)	(22.5%)	(38.5%)	(1.09–1.12)	(23.1%)	(41.2%)	(0.95–1.12)	(38.1%)	(26.9%)	(0.67–1.36)	(25.1%)	(53.8%)	(0.98–1.01)
**Admission type**															
Medical	50,186	12,250	1	134,020	52,703	1	3,072	1,413	1	327	119	1	51,222	30,737	1
	(21.9%)	(24.4%)	-	(24.4%)	(39.3%)	-	(25.4%)	(46.0%)	-	(21.8%)	(36.4%)	-	(19.5%)	(60.0%)	-
Surgical	66,002	10,917	0.89 **	170,728	40,344	0.64 **	4,213	1,261	0.76 **	506	120	0.9	74,647	32,468	0.84 **
	(28.9%)	(16.5%)	(0.87–0.92)	(31.1%)	(23.6%)	(0.63–0.65)	(34.8%)	(29.9%)	(0.7–0.83)	(33.7%)	(23.7%)	(0.67–1.19)	(28.4%)	(43.5%)	(0.83–0.86)
Other	112,499	19,841	0.96 **	244,217	89,688	0.93 **	4,822	1,784	0.97	667	162	0.87	136,795	70,866	1.04 **
	(49.2%)	(17.6%)	(0.94–0.99)	(44.5%)	(36.7%)	(0.92–0.94)	(39.8%)	(37.0%)	(0.90–1.05)	(44.5%)	(24.3%)	(0.67–1.14)	(52.1%)	(51.8%)	(1.03–1.06)
**Admission source**															
Emergency	145,011	26,726	1	331,724	112,778	1	8,829	3,344	1	1,041	281	1	152,676	78,627	1
	(63.4%)	(18.4%)	-	(60.4%)	(34.0%)	-	(72.9%)	(37.9%)	-	(69.4%)	(27.0%)	-	(58.1%)	(51.5%)	-
Planned	47,451	10,704	1.12 **	140,389	45,852	0.91 **	1,701	682	0.99	301	75	0.77	58,515	34,485	1.00
	(20.7%)	(22.6%)	(1.08–1.15)	(25.6%)	(32.7%)	(0.89–0.92)	(14.0%)	(40.1%)	(0.90–1.08)	(20.1%)	(24.9%)	(0.57–1.04)	(22.3%)	(58.9%)	(0.98–1.01)
Other	36,225	5,578	1.03 *	76,852	24,105	0.93 **	1,577	432	0.8 **	158	45	1.18	51,473	20,959	0.87 **
	(15.8%)	(15.4%)	(1.00–1.06)	(14.0%)	(31.4%)	(0.92–0.94)	(13.0%)	(27.4%)	(0.73–0.89)	(10.5%)	(28.5%)	(0.86–1.63)	(19.6%)	(40.7%)	(0.86–0.88)
**Length of stay**															
1–2 days	50,380	11,931	1	136,777	48,274	1	3,293	1,435	1	343	128	1	52,808	30,741	1
	(22.0%)	(23.7%)	-	(24.9%)	(35.3%)	-	(27.2%)	(43.6%)	-	(22.9%)	(37.3%)	-	(20.1%)	(58.2%)	-
3–5 days	60,030	11,551	0.96 **	152,240	46,593	0.94 **	3,272	1,207	0.95	383	107	0.83	65,695	34,274	1.00
	(26.2%)	(19.2%)	(0.93–0.98)	(27.7%)	(30.6%)	(0.93–0.95)	(27.0%)	(36.9%)	(0.88–1.03)	(25.5%)	(27.9%)	(0.63–1.08)	(25.0%)	(52.2%)	(0.98–1.02)
5–10 days	55,974	9,705	1.00	127,815	39,958	1.02 *	2,785	947	0.98	364	92	0.84	64,414	31,434	1.05 **
	(24.5%)	(17.3%)	(0.97–1.03)	(23.3%)	(31.3%)	(1.00–1.03)	(23.0%)	(34.0%)	(0.90–1.08)	(24.3%)	(25.3%)	(0.62–1.13)	(24.5%)	(48.8%)	(1.03–1.07)
10+ days	62,303	9,821	1.07 **	132,133	47,910	1.24 **	2,757	869	1.03	410	74	0.64 *	79,747	37,622	1.15 **
	(27.2%)	(15.8%)	(1.03–1.10)	(24.1%)	(36.3%)	(1.22–1.26)	(22.8%)	(31.5%)	(0.93–1.13)	(27.3%)	(18.0%)	(0.46–0.90)	(30.4%)	(47.2%)	(1.13–1.17)
Total admissions	228,687	43,008		548,965	182,735		12,107	4,458		1500	401		262,664	134,071	
		(18.8%)			(33.2%)			(36.8%)			(26.7%)			(51.0%)	
Total patients	76,666	23,245		204,332	95,077		4,199	2,043		550	214		95,874	63,286	

N: Total number of inspected admissions (denominator); n: Number of admissions with non-recorded condition (numerator); IR (%): Incidence rate (per 100 admissions).

Incidence rate ratios (IRR) and related confident intervals (CI) were obtained using a Poisson model.

Admissions with missing in any covariate were excluded.

No admissions existed for patients with hepatitis who were aged 17 years and under or 90 years and over.

* Significant at 5%

** significant at 1%.

Discrepancy incidents were lower among females for most of the chronic conditions, with the largest gender difference observed for hypertension (21%). For older patients, hepatitis was more accurately recorded, while diabetes and smoking conditions were less likely to be documented than for younger patients. Patients’ socio-economic status had either no or an inconsistent effect on coding completeness. Patients who underwent surgery during hospitalisation were up to 36% more likely to have their chronic conditions recorded compared to the medical patients. The effect of admission source was inconsistent across chronic conditions; emergency admitted patients had a lower discrepancy incidence rate for diabetes, but a higher rate for smoking compared to planned admissions. A similar inconsistent pattern was evident for the effect of length of stay on completeness of recording chronic conditions (Table 1).

The incidence of non-recorded chronic conditions was significantly higher in private hospitals across all conditions. For the most common conditions of diabetes, smoking and hypertension, the excess likelihood of discrepancy in recording morbidities within private hospitals ranged between 15% and 22%. Significantly higher inconsistency rates of 60% and 201% in clinical records were found for patients with hepatitis and HIV. Rural hospitals tended to have up to an 8% lower discrepancy incident rate in recording the top three most common chronic conditions; no difference was observed between metropolitan and rural hospitals in recording HIV and hepatitis. Among public hospitals, smaller hospitals had higher discrepancy incidence rates mainly for the top three most common conditions, compared to large principal referral hospitals. The highest gaps of at least 90% and the lowest gaps of at most 20% were found in recording diabetes and hypertension respectively (Table 2).

**Table 2:** Admissions, discrepancies and associated incidence rates and rate ratios, stratified by comorbidity and hospital characteristics.

Characteristics	Diabetes			Smoking			Hepatitis			HIV			Hypertension		
	N (%)	n (IR%)	IRR (95% CI)	N (%)	n (IR%)	IRR (95% CI)	N (%)	n (IR%)	IRR (95% CI)	N (%)	n (IR%)	IRR (95% CI)	N (%)	n (IR%)	IRR (95% CI)
**Quartile of coded conditions**															
1^st^ (least comprehensive)	35,043	15,185	1	149,250	63,747	1	1,640	1,126	1	234	91	1	44,550	36,267	1
	(15.3%)	(43.3%)	-	(27.2%)	(42.7%)	-	(13.5%)	(68.7%)	-	(15.6%)	(38.9%)	-	(17.0%)	(81.4%)	-
2^nd^	63,149	14,136	0.61 **	176,634	55,959	0.74 **	3,706	1,488	0.62 **	495	158	0.88	72,341	41,695	0.71 **
	(27.6%)	(22.4%)	(0.59–0.62)	(32.2%)	(31.7%)	(0.73–0.75)	(30.6%)	(40.2%)	(0.58–0.68)	(33.0%)	(31.9%)	(0.67–1.14)	(27.5%)	(57.6%)	(0.7–0.72)
3^rd^	36,683	5,511	0.41 **	76,590	22,883	0.68 **	2,085	656	0.51 **	241	59	0.74	41,177	19,698	0.59 **
	(16.0%)	(15.0%)	(0.40–0.43)	(14.0%)	(29.9%)	(0.67–0.69)	(17.2%)	(31.5%)	(0.46–0.56)	(16.1%)	(24.5%)	(0.53–1.04)	(15.7%)	(47.8%)	(0.57–0.60)
4^th^ (most comprehensive)	93,812	8,176	0.23 **	146,491	40,146	0.61 **	4,676	1,188	0.43 **	530	93	0.58 **	104,596	36,411	0.43 **
	(41.0%)	(8.7%)	(0.23–0.24)	(26.7%)	(27.4%)	(0.60–0.62)	(38.6%)	(25.4%)	(0.40–0.48)	(35.3%)	(17.5%)	(0.42–0.80)	(39.8%)	(34.8%)	(0.42–0.44)
**Other chronic condition**															
No	117,074	28,501		405,066	140,267	1	4,867	2,155	1	759	203	1	134,981	78,592	1
	(51.2%)	(24.3%)		(73.8%)	(34.6%)	-	(40.2%)	(44.3%)	-	(50.6%)	(26.7%)	-	(51.4%)	(58.2%)	-
Yes	111,613	14,507	0.55 **	143,899	42,468	0.87 **	7,240	2,303	0.74 **	741	198	1.05	127,683	55,479	0.76 **
	(48.8%)	(13.0%)	(0.54–0.56)	(26.2%)	(29.5%)	(0.86–0.88)	(59.8%)	(31.8%)	(0.70–0.79)	(49.4%)	(26.7%)	(0.86–1.29)	(48.6%)	(43.5%)	(0.75–0.76)
**Non-coded other chronic condition** ^#^															
No	78,287	8,886	1	93,119	27,077	1	5,619	1,651	1	514	129	1	101,068	42,294	1
	(54.5%)	(11.4%)	-	(56.2%)	(29.1%)	-	(59.8%)	(29.4%)	-	(56.4%)	(25.1%)	-	(64.5%)	(41.8%)	-
Yes	65,356	19,361	2.51 **	72,556	34,521	1.56 **	3,785	1,912	1.67 **	398	152	1.49 **	55,637	37,042	1.53 **
	(45.5%)	(29.6%)	(2.45–2.58)	(43.8%)	(47.6%)	(1.54–1.59)	(40.2%)	(50.5%)	(1.56–1.78)	(43.6%)	(38.2%)	(1.17–1.89)	(35.5%)	(66.6%)	(1.50–1.55)
**Hospitals type**															
Public	195,508	35,109	1	447,133	144,043	1	11,764	4,256	1	1,457	376	1	216,517	104,597	1
	(85.5%)	(18.0%)	-	(81.5%)	(32.2%)	-	(97.2%)	(36.2%)	-	(97.1%)	(25.8%)	-	(82.4%)	(48.3%)	-
Private	33,179	7,899	1.15 **	101,832	38,692	1.17 **	343	202	1.60 **	43	25	3.01 **	46,147	29,474	1.22 **
	(14.5%)	(23.8%)	(1.12–1.19)	(18.5%)	(38.0%)	(1.15–1.19)	(2.8%)	(58.9%)	(1.36–1.87)	(2.9%)	(58.1%)	(1.88–4.83)	(17.6%)	(63.9%)	(1.20–1.24)
**Local health district**															
Metropolitan	160,938	30,534	1	384,575	130,888	1	9,522	3,490	1	1,289	338	1	183,974	94,687	1
	(70.4%)	(19.0%)	-	(70.1%)	(34.0%)	-	(78.6%)	(36.7%)	-	(85.9%)	(26.2%)	-	(70.0%)	(51.5%)	-
Rural	67,749	12,474	0.92 **	164,390	51,847	0.96 **	2,585	968	1.04	211	63	1.20	78,690	39,384	0.94 **
	(29.6%)	(18.4%)	(0.90–0.94)	(29.9%)	(31.5%)	(0.95–0.97)	(21.4%)	(37.4%)	(0.96–1.12)	(14.1%)	(29.9%)	(0.87–1.65)	(30.0%)	(50.0%)	(0.93–0.96)
**Peer hospital groups (public only)** ^#^															
Principal referral (A1)	100,761	17,244	1	221,444	71,324	1	7,853	2,737	1	1,219	295	1	112,821	50,790	1
	(51.5%)	(17.1%)	-	(49.5%)	(32.2%)	-	(66.8%)	(34.9%)	-	(83.7%)	(24.2%)	-	(52.1%)	(45.0%)	-
Major metro/non-metro (B)	60,601	11,054	2.49 **	154,621	45,890	1.24 **	2,840	1,026	1.05	187	59	1.4	65,504	33,778	1.14 **
	(31.0%)	(18.2%)	(2.13–2.92)	(34.6%)	(29.7%)	(1.14–1.34)	(24.1%)	(36.1%)	(0.93–1.17)	(12.8%)	(31.6%)	(0.90–2.19)	(30.3%)	(51.6%)	(1.06–1.22)
District group 1 (C1)	18,315	3,297	1.89 **	40,089	13,157	1.22 *	544	235	1.23 *	25	8	1.65	19,879	10,181	1.14 *
	(9.4%)	(18.0%)	(1.47–2.43)	(9.0%)	(32.8%)	(1.01–1.48)	(4.6%)	(43.2%)	(1.03–1.46)	(1.7%)	(32.0%)	(0.74–3.66)	(9.2%)	(51.2%)	(1.02–1.27)
District group 2 (C2)	15,831	3,514	2.13 **	30,979	13,672	1.58 **	527	258	1.29 **	26	14	2.61 *	18,313	9,848	1.19 **
	(8.1%)	(22.2%)	(1.71–2.66)	(6.9%)	(44.1%)	(1.35–1.86)	(4.5%)	(49.0%)	(1.08–1.54)	(1.8%)	(53.8%)	(1.22–5.57)	(8.5%)	(53.8%)	(1.08–1.31)

N: Total number of inspected admissions (denominator); n: Number of admissions with non-recorded condition (numerator); IR (%): Incidence rate (per 100 admissions).

Incidence rate ratios (IRR) and related confident intervals (CI) were obtained using a Poisson model.

Admissions with missing in any covariate were excluded.

^**#**^ Numbers and estimates were calculated using a subset of admissions according to the covariate definition.

* Significant at 5%

** significant at 1%.

A higher number of recorded comorbidities at hospital admission decreased the likelihood of non-recorded chronic conditions by at least 40%. Each chronic condition (except HIV) was more likely (at least 13%) to be recorded if the patient had any other recorded chronic conditions. The likelihood of not recording a chronic condition could increase in the omission of the recording of the other four conditions by at least 49% (Table 2).

### 3.2 Trend analysis

**Fig 1:**
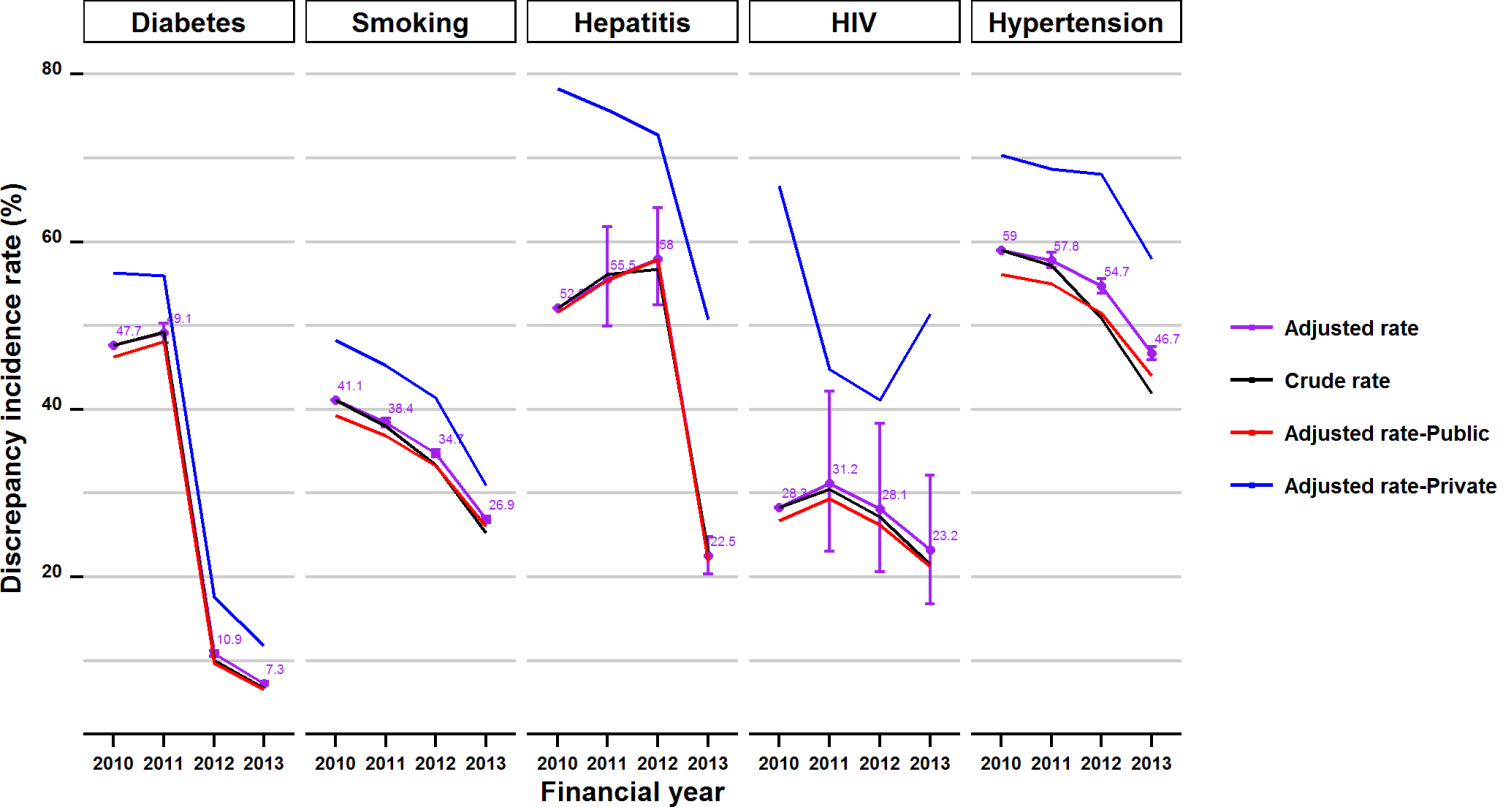
Crude and adjusted trends (overall and stratified by hospital type) of discrepancy incidence rates for the five chronic conditions over the study period.

As depicted in Fig 1, discrepancy incidents for all examined chronic conditions declined over the four-year period (2010–2013 FY). The sharpest drop of close to 85% was observed for diabetes (adjusted rates of 47.7% in 2010 vs. 7.3% in 2013). For hepatitis the adjusted rate increased by 6% in 2012, reaching 58%, then markedly dropped by over 60% in 2013, with a total drop of 56% over the study period. Incidence rates for smoking and hypertension notably decreased by 35% and 20% respectively, but rates were unchanged for HIV with a non-significant drop of 18%. The discrepancy incidence rate in public hospitals remained lower than the rate in the private hospitals over the study period for all the chronic conditions. Up to 4% larger drops were observed for diabetes and hypertension rates in public versus private hospitals; whereas the drop in the discrepancy rate for smoking was 10% larger in private compared to public hospitals. The observed differences in trends for hepatitis and HIV discrepancy rates were not significant.

### 3.3 Inter-hospital variation

Adjustment for patient and admission characteristics explained much of the observed inter-hospital variations in discrepancy incidents, as seen by large drops in the VPC from the model with no adjustment to models with patient and admission factors across all chronic conditions (Table 3). However, a noticeable proportion of between 0.9% (for HIV) and 9.4% (for smoking) of all variations was still attributable to hospital and associated factors. Hospital characteristics (rurality and peer groups), partly explained the inter-hospital variation, leaving up to 7% of unexplained variation that was associated with individual hospital characteristics (unseen factors).

**Table 3:** Inter-hospital variation and associated statistics for discrepancies among 80 NSW public hospitals.

Models	Diabetes			Smoking			Hepatitis			HIV			Hypertension		
	Variance (Reduction)	MIRR	VPC (IQR)	Variance (Reduction)	MIRR	VPC (IQR)	Variance (Reduction)	MIRR	VPC (IQR)	Variance (Reduction)	MIRR	VPC (IQR)	Variance (Reduction)	MIRR	VPC (IQR)
**Intercept only**	0.047	1.23	92.8% (83.3–96.3)	0.112	1.37	99.3% (97.9–99.7)	0.115	1.38	16.1% (7.1–46.9)	0.035	1.19	39% (15.2–68.8)	0.041	1.21	97.1% (92.5–98.6)
**Patient factors**	0.046 (1.4%)	1.23	11% (2.3–40.5)	0.113 (-0.8%)	1.38	53.4% (17–87.2)	0.116 (-1.3%)	1.38	7.3% (4.1–15.7)	0.027 (21.9%)	1.17	4.3% (1.9–11.7)	0.042 (-2.3%)	1.21	30.7% (7.6–68.9)
**Patient and admission factors**	0.039 (17.2%)	1.21	1.7% (0.8–4.7)	0.092 (18.4%)	1.33	9.4% (3.9–24.6)	0.089 (22.2%)	1.33	3.2% (2.3–4.6)	0.02 (42.5%)	1.14	0.9% (0.7–1.8)	0.039 (6.2%)	1.20	4.9% (2.2–13.0)
**Patient, admission and hospital factors**	0.029 (38.0%)	1.18	1.3% (0.6–3.4)	0.071 (37.0%)	1.29	6.9% (3.0–18.8)	0.061 (46.7%)	1.26	2.0% (1.5–3.0)	0.013 (61.2%)	1.12	0.6% (0.5–1.2)	0.032 (21.8%)	1.19	4.0% (1.8–10.7)

MIRR: Median incidence rate ratio; VPC: Variance partition coefficient (hospital level variance divided by total variance); IQR: Inter-quartile range.

Inter-hospital (hospital level) variances were obtained using Poisson mixed models.

Variance reduction is the proportional decrease in the estimated hospital level variances from the extended model against the intercept model.

Due to varying VPC in Poisson models, median of conditional VPCs over existing covariate values were reported here. Conditional VPCs were derived using the direct calculation method [31].

MIRR was calculated as $exp(0.954\times \sqrt{variance})$.

Overall, the presence of smoking or hepatitis in a patient admitted to a hospital with high discrepancy rates, was up to 33% (MIRR = 1.33, adjusted for patient and admission characteristics) more likely not to be recorded, than had the admission been to a hospital with lower discrepancy rates. A smaller gap of close to 20% was observed for diabetes and hypertension; followed by 14% for HIV, the condition most robust to hospital characterises (Table 3). According to proportional variance reductions, case-mix factors explained between 22% (the lowest for hypertension) and 61% (the highest for HIV) of inter-hospital variations. Most of this was explained by hospital and admission factors as opposed to patient demographics, as inclusion of them largely decreased the estimated variances across all chronic conditions. In particular, of all inter-hospital variations, between 8% (for hypertension) and 21% (for HIV) was further explained by admission factors. An additional explained variation of 16% (for hypertension) to 25% (for hepatitis) was obtained following the inclusion of hospital factors (Table 3).

**Fig 2:**
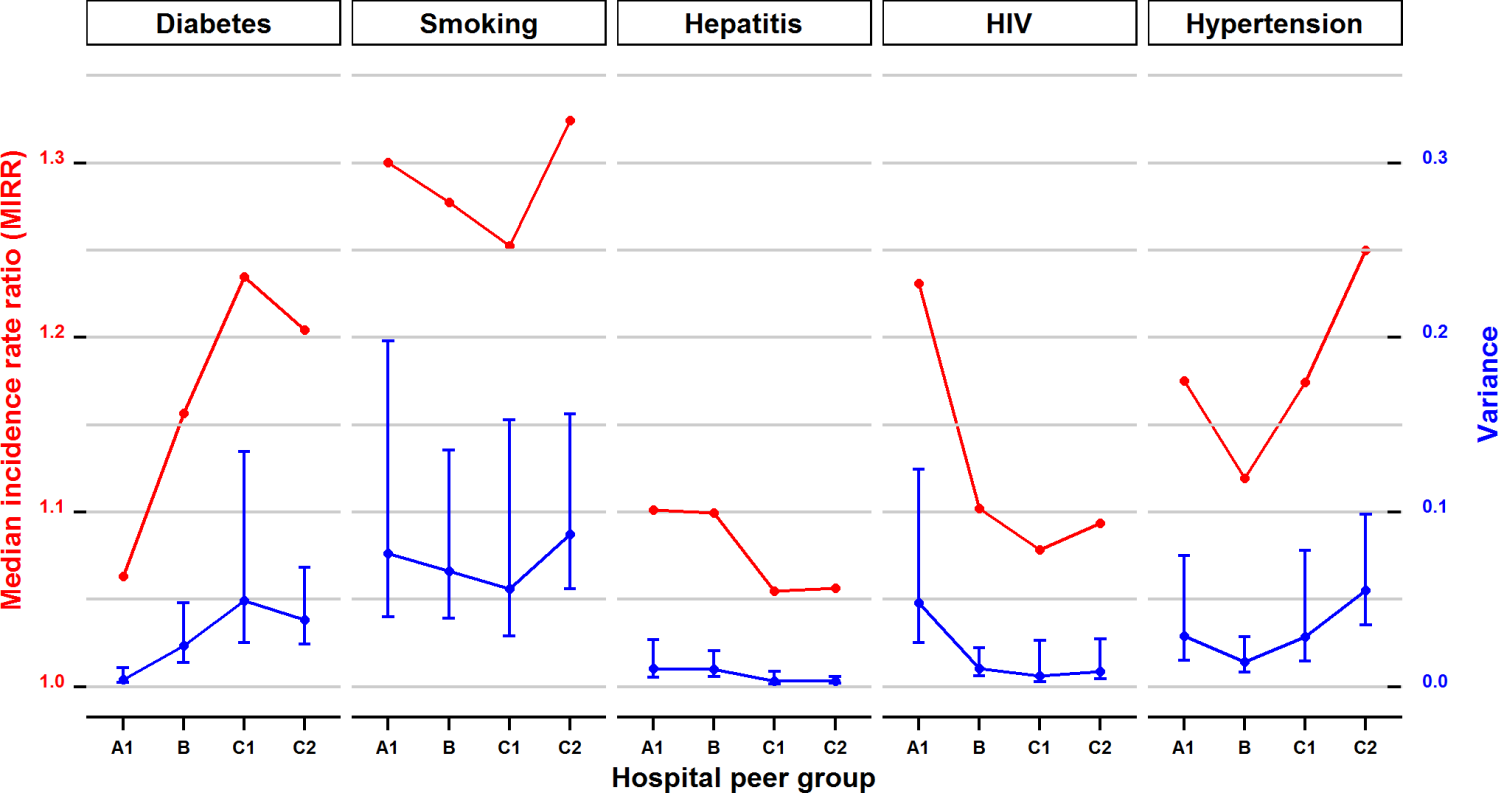
Inter-hospital variance and associated median incidence rate ratio of discrepancy incidents for the five chronic conditions among 80 NSW public hospitals stratified by hospital peer groups.

The extent to which inter-hospital variations and likelihood gaps in recording chronic conditions were influenced by hospital size varied across chronic conditions (Fig 2). Large principal referral hospitals (A1) tended to have smaller variations in recording diabetes, but higher variations for HIV compared to the smaller district hospitals (C1 and C2). The observed variations in recording diabetes translated to a 9% gap among principal referral hospitals compared to a 17% gap in the group with the smallest district hospitals (C2); the relevant numbers for HIV were 24% and 9% respectively. No consistent pattern or considerable difference was observed among hospital groups in recording other chronic conditions.

**Fig 3:**
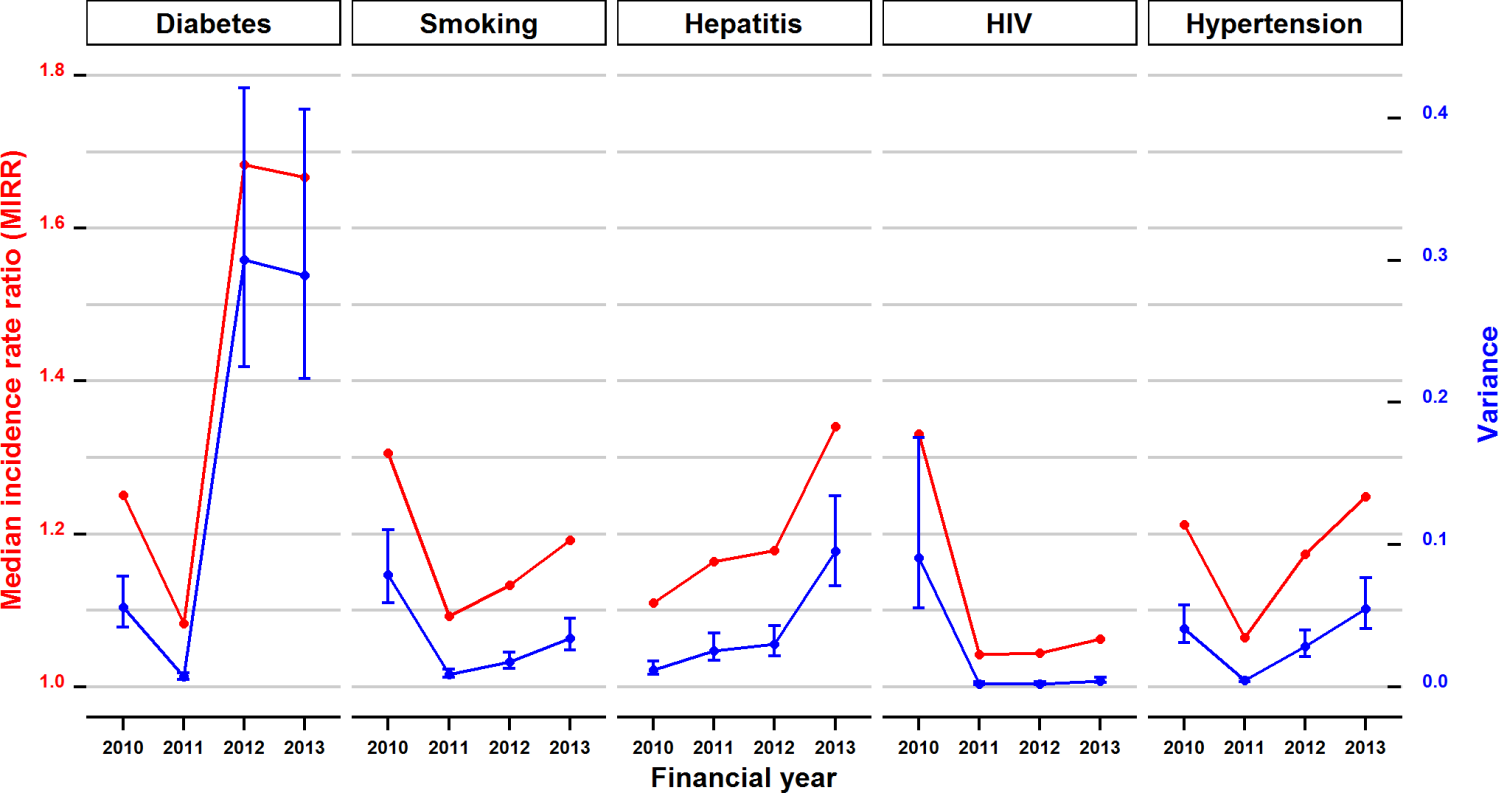
Trends of inter-hospital variance and associated median incidence rate ratio for discrepancy incidents of five chronic conditions among 80 NSW public hospitals over the study period.

The inter-hospital variations in discrepancy incidence rates varied over time for all chronic conditions. In particular, there were greater differences in the coding of diabetes in the second half of the study period compared to the first half (Fig 3). The gap of at most 25% for diabetes in the first period increased to over 65%. An increase from 10% to 34% was also evident for the coding of hepatitis. The trends in the variation of the other three conditions noticeably decreased in 2011 and subsequently either remained stable or began to increase over the next three years.

Hospitals with lower discrepancy rates in recording diabetes tended to also have lower discrepancy rates in the recording of smoking since a significant correlation between deviations from the average (estimated hospital-specific intercept) in the two conditions was observed across 80 hospitals. A similar pattern among hospitals was also observed for the recording of hepatitis and smoking as well as for hypertension and HIV; see S2 Table.

## Discussion

### Key findings

This large population-based study using NSW Ministry of Health hospital admissions linked datasets over a four-year period identified the non-recorded incidence rates of five chronic conditions as varying between 19% (for diabetes) and 51% (for hypertension). Except for HIV, the adjusted discrepancy incidence rates for all examined chronic conditions declined considerably, ranging from 20% for hypertension to 80% for diabetes over the four-year period to July 2014. Admission records from private hospitals and smaller public hospitals had higher discrepancy incidents compared to their counterparts. Variability among public hospitals was responsible for 1% to 9% of variation in adjusted discrepancy incidence rates for the five chronic conditions, leading to between 14% and 33% discrepancy rate differences. Seven per cent of the variation remained unexplained after adjusting for hospital characteristics. The inter-hospital variation changed over time, with the increase most noticeable for diabetes. Hospital size had an inconsistent effect on inter-hospital discrepancy differences across the conditions.

### Discrepancy incidence rates and trends

Completeness in recording chronic morbidities and agreement among different sources of morbidity data have been investigated in Australia and elsewhere. The identified 19% incompleteness in the coding of diabetes in the NSW hospital administrative data was in the range of the previously reported rates of at most 13% [17–19, 36] and 26% [7] when clinical charts and self-reported information, respectively were used as the reference. The large drop in discrepancy incidence rates for diabetes from over 47% in the first half of our study period to 10% or less in the last two years coincided with the change in rules governing the coding of diabetes as comorbidity in hospital data. In general, according to the Australian ICD standard for documenting additional diagnoses in clinical charts, only those conditions affecting the patient’s care management or treatment within that admission are required to be coded in hospital administrative datasets [5]. Therefore, diagnoses that relate to an earlier admission, and which have no effect on the current admission, are not required to be coded. The cause and effect relationship requirement for coding purposes between diabetes and the patient’s care, which was applied during the 2010 to 2012 period, was lifted in July 2012 [27]. Such changes reportedly influenced diabetes prevalence estimates based on administrative data [22, 37] and the occurrence of discrepancies as demonstrated in this study. Our findings reflected the influence of the change in standards that lead to reduced subjectivity associated with coding at the coder level. In particular, it revealed the potential improvement in recording (documented) diabetes by coders versus the lack of documentation of diabetes in clinical charts by clinicians [4].

A lower discrepancy rate of 19% in coding smoking status was observed in the UK administrative datasets [19], compared to the 33% identified in this study and the 41% reported recently from NSW APDC datasets [7]. Inclusion of tobacco related service use in the UK study could have contributed to lower inconsistency, while identification of ex-smokers and tobacco related injuries in our study compared to the recent Australian research may have resulted in better completeness rates identified in this study.

The observed 51% discrepancy rate for hypertension was almost double that seen when clinical charts were the reference [17, 19], but was lower than the rate of 69% obtained using patients’ self-report [7]. Compared to other reports, we applied the narrowest ICD codes in case identification, disregarding cases with renal, brain or pregnancy complications caused by hypertension which could have resulted in different completeness rates.

For the rare conditions of hepatitis and HIV, our study benefited from a large state-wide cohort, providing more reliable discrepancy rates (of 37% and 27%) compared to other reports (zero to 33%) limited by small sample size [17, 18, 38, 39]. We found noticeably high inconsistencies in coding morbidities that are either life threating or can cause severe complications, as is the case for HIV, which is listed among the most important risk factors of mortality in risk adjustment methods [23, 24].

In addition to changes in coding standards and varying case identification methods noted above, systematic changes that affect coding practices as well as the method of verification to identify non-recorded comorbidities in hospital data may also have contributed to the differences in reported discrepancies. The observed decreasing trends for all conditions, particularly within public hospitals, can be associated with the introduction of activity based funding in 2011 in Australia [21, 40] as it previously resulted in increases in the recording of secondary diagnoses and procedures [12, 41] in Europe. Responses to shortfalls in staffing and training of clinical coders prior to our study period [42] could have contributed to the temporal reduction in discrepancies as observed elsewhere [8, 39].

The current findings indicate higher discrepancy rates compared to studies conducted using clinical chart review, regarded as the gold standard [17, 19, 38], to ascertain the presence of chronic conditions but lower rates than studies using primary carer provider or patient survey information [7, 18]. Having higher rates compared to clinical chart-based studies could be due to inclusion of non-recorded conditions as true non-documented conditions in our rates. Despite the potential to report false positive rates (falsely recorded conditions) in studies measuring agreement between hospital data and external references, comparison of hospital data with clinical charts indeed focuses on discrepancy in the coding conditions that were documented. Using other references such as survey based information may still overlook non-recorded conditions, and not resolve problems of high subjectivity and variation due to lack of unique governing standards in documentation. The employed internal references developed and applied in this study enabled the capture of all non-recorded conditions regardless of whether they were documented within one environment and governed by a unique set of rules. Although the effect of temporal data accumulation was not determined, using a prospective design enabled us to directly estimate the amount of discrepancy which can be eliminated through data accumulation over time. The demonstrated increase in accuracy of hospital data through temporal data accumulation [7, 36] also support the utilisation of internal references within this setting. The very low false positive rates of less than 2% in administrative datasets [7, 18] for most of the conditions investigated gives further credence for the reliability of our internal references (index admission being true positive) made possible with data linkage and lends credibility to our sole focus on non-recorded comorbidities.

We echoed other research findings of higher discrepancies among private hospitals compared to public hospitals [7, 8, 17, 18]. The role of clinical coding in funding public hospitals could result in improved accuracy in public hospital datasets [12, 41]. Our finding that rural hospitals tended to have more accurate recorded conditions was consistent with US results [8, 20] but contradicted previous Australian findings [17]. However, the significantly higher discrepancy rates in smaller versus larger public hospitals were consistent with previous Australian findings [7, 17]. A tendency to record more comorbidity at larger hospitals, reflecting the presentation of severely ill patients with multiple conditions was positively associated with better accuracy in administrative datasets [7, 13].

### Inter-hospital variation

Variation in performance [43, 44], quality and safety [45, 46] and service usage [47] indicators among acute care providers in NSW and elsewhere has been identified. Taking into account patient and admission differences, notable inter-hospital variability in discrepancy incidence rates was evident among 80 NSW public hospitals. A third of our adjusted inter-hospital variation (0.9% to 9.4%) was explained by hospital size and location. Our results were comparable to Lujic et al. [7] who reported a slightly higher variation (2% to 13%) among similar hospitals. Differences in the modelling scheme, measurement and adjustments would have contributed to the results.

Larger variation in recording hypertension and smoking than diabetes were consistent with previous findings [7]. The contribution of case mix adjustment in explaining inter-hospital variation differed across chronic conditions with the highest for HIV and hepatitis and the lowest for hypertension. No comparative data exist for examining the variability in recording hepatitis and HIV conditions. These findings highlighted the potential biases, caused by discrepancies in coding, for care provider comparisons and funding based on risk adjustment methods, in particular those using hypertension, as has been addressed [7] and evaluated [13] elsewhere.

Discrepancy rates as well as inter-hospital variation varied over time and were affected by hospital size. Despite the observed drop in the discrepancy rate for diabetes, a significant increase in the related inter-hospital variation over the second half of the study period was evident, perhaps reflecting differences in the method and speed of adoption of modified coding rules for diabetes [27]. The timing and level of adaptation to new standards among hospitals can introduce larger variation at least in the short term. The introduction of activity-based funding might also have contributed to the overall increasing trend of variation observed from 2011 for the other conditions [40].

At the patient level, discrepancy rates for each condition were inversely associated with the number of recorded conditions and, in particular if the recorded comorbidities included one of our five chronic conditions. At the higher level, hospitals with good coding practice for one condition tended to do well with others. These findings reemphasise the importance of individual hospital responsibilities and characteristics. Engagement of coders in diverse roles, higher staffing and lower throughput, training and professional development and interaction with clinicians are among the effective organisational factors aimed at enhancing clinical data quality [8, 9]. Enhancement and standardisation of training and rotation of coders between hospitals have also reduced variation at coder level [39].

### Implications

Our study raises several important policy implications. Firstly, despite advancement in adjustment methods to ascertain fair comparison and funding strategies, the significant non-random inconsistencies in the administrative dataset are likely to lead to disproportionate conclusions. Minimising discrepancies or at least controlling for hospitals level factors through modelling or stratification will facilitate optimal decision making. Secondly, in the absence of routine utilisation of clinical chart review, the use of temporal accumulation of morbidity information within administrative datasets to measure discrepancy and construct informed risk adjustment is feasible, as demonstrated by this study. Thirdly, defining quality characteristics for administrative data and routinely monitoring the quality indicators over time would allow better understanding of the effectiveness of system changes, such as documentation and recording standards, and highlight areas for improvement and subsequent actions [48, 49]. Lastly, systematic knowledge enhancement and engagement among hospital administrators, clinicians, coders and researchers within health service domain for recording quality improvement and reimbursement purposes should be formalised.

### Strengths and limitations

This study benefited from its design, using a large population based dataset to access all admissions in all acute hospitals within the most populated health jurisdiction in Australia to explore for the first time trends in coding discrepancy rates. This study benefited from data linkage at patient level and a prospective longitudinal design. The design enabled the exclusion of any pre-diagnostic admissions, to eliminate the risk of any overestimation in discrepancy rates, and combined with a restricted set of criteria and follow-up period minimised any false positives due to error or post treatment. The proposed design developed and employed internal references based on routinely collected data that could readily be used for real time monitoring of clinical coding practices and improvement through longitudinal data accumulation and dynamic indexing.

We may have under-reported the total discrepancies in the absence of an external reference for measuring false positive rates. However, clinical chart review on randomly sampled cases although useful, is limited to the extent that it relies on comprehensive documentation of all comorbidities. Variation analysis was limited to public hospitals as determined by data availability; the analyses of hospital-specific admissions from private hospitals could provide addition insight. Despite the essential role of time in our design, the effect size of data accumulation as well as time between multiple admissions was not quantified and is therefore an area for further research. Models incorporating a coder’s related characteristics including staffing, rotation, experience and training, coding parameters such as rules governing the documentation and coding of a condition and unseen admission factors including principal diagnoses may better explain differences. Distinguishing contribution to discrepancy from incomplete documentation of morbidity in clinical chart versus incomplete coding of documented conditions in hospital administrative datasets would be very informative for targeted actions. Conducting a controlled trail or comparing patients’ records across transfers could provide valuable insight of documentation versus coding contributions in discrepancies. Inclusion of changes in the rules governing recording practices in the modelling might also provide more evidence on the effect of system-wide changes and further highlight potential areas for improvement.

## Conclusion

Chronic conditions are recorded inconsistently in hospital administrative datasets, and hospitals, individually as well as grouped by characteristics, contribute to the observed incidence and variation in discrepancies. Consequently, case-mix adjustments for provider comparison and funding purposes could be biased because of coding incompleteness and associated discrepancy patterns across hospitals. While examination of non-recording patterns associated with hospital characteristics through modelling or stratification for risk-adjustment purposes could potentially minimise bias, longitudinal accumulation of clinical information at patient level through data linkage combined with refinement of clinical coding systems and standardisation of documentation across hospitals would enhance accuracy of routinely collected datasets and the related validity of case-mix adjustment.

## Supporting Information

S1 Table Pair-wise correlation of hospital performance in recording chronic conditions. (DOCX)

S2 Table Discrepancy incidences using different settings. Number of admissions included in discrepancy incidence rate calculation for each of the five chronic conditions using different follow-up periods and buffers.
(DOCX)
