# Supplementary material for: Incidence and Variation of Discrepancies in Recording Chronic Conditions in Australian Hospital Administrative Data
Source: PLoS One. 2016 Jan 25;11(1):e0147087. doi: 10.1371/journal.pone.0147087 (PMC4726608; doi:10.1371/journal.pone.0147087)
Supplement: S1 Table — (DOCX) [file pone.0147087.s001.docx]

S1 Table. Pair-wise correlation of hospital performance in recording chronic conditions.

| **Chronic condition** | **Diabetes** | **Smoking** | **Hepatitis** | **HIV** | **Hypertension** |
| --- | --- | --- | --- | --- | --- |
| **Diabetes** | 1.00 |  |  |  |  |
| **Smoking** | 0.31** | 1.00 |  |  |  |
| **Hepatitis** | 0.00 | 0.24* | 1.00 |  |  |
| **HIV** | 0.03 | 0.06 | 0.05 | 1.00 |  |
| **Hypertension** | 0.06 | 0.06 | -0.02 | 0.32* | 1.00 |

Pearson correlation coefficients are between hospital-specific intercept estimates obtained from Poison mixed models.

* Significant at 5%; ** significant at 1%.
