# Supplementary material for: Incidence and Variation of Discrepancies in Recording Chronic Conditions in Australian Hospital Administrative Data
Source: PLoS One. 2016 Jan 25;11(1):e0147087. doi: 10.1371/journal.pone.0147087 (PMC4726608; doi:10.1371/journal.pone.0147087)
Supplement: S2 Table — Number of admissions included in discrepancy incidence rate calculation for each of the five chronic conditions using different follow-up periods and buffers. (DOCX) [file pone.0147087.s002.docx]

S2 Table. Discrepancy incidences using different settings. Number of admissions included in discrepancy incidence rate calculation for each of the five chronic conditions using different follow-up periods and buffers.

| **Chronic condition** | **Outcome** | **In-between and follow-up admissions without buffer within** | | | |  | **In-between and follow-up admissions**  **with buffer within** | | |
| --- | --- | --- | --- | --- | --- | --- | --- | --- | --- |
|  |  | **No follow-up** | **3 months** | **6 months** | **12 months** |  | **3 months*** | **6 months** | **12 months** |
| **Diabetes** | **N** | 217,172 | 230,418 | 234,590 | 239,499 |  | 229,685 | 233,437 | 236,476 |
|  | **n** | 30,785 | 44,031 | 48,203 | 53,112 |  | 43,298 | 47,050 | 50,089 |
|  | **IR (%)** | 14.2% | 19.1% | 20.5% | 22.2% |  | 18.9% | 20.2% | 21.2% |
|  |  |  |  |  |  |  |  |  |  |
| **Smoking** | **N** | 482,278 | 558,126 | 582,608 | 615,318 |  | 553,618 | 575,277 | 595,057 |
|  | **n** | 113,337 | 189,185 | 213,667 | 246,377 |  | 184,677 | 206,336 | 226,116 |
|  | **IR (%)** | 23.5% | 33.9% | 36.7% | 40.0% |  | 33.4% | 35.9% | 38.0% |
|  |  |  |  |  |  |  |  |  |  |
| **Hepatitis** | **N** | 10,962 | 12,731 | 13,465 | 14,141 |  | 12,519 | 13,092 | 13,209 |
|  | **n** | 3,117 | 4,886 | 5,620 | 6,296 |  | 4,674 | 5,247 | 5,364 |
|  | **IR (%)** | 28.4% | 38.4% | 41.7% | 44.5% |  | 37.3% | 40.1% | 40.6% |
|  |  |  |  |  |  |  |  |  |  |
| **HIV** | **N** | 1,387 | 1,546 | 1,622 | 1,770 |  | 1,539 | 1,602 | 1,705 |
|  | **n** | 264 | 423 | 499 | 647 |  | 416 | 479 | 582 |
|  | **IR (%)** | 19.0% | 27.4% | 30.8% | 36.6% |  | 27.0% | 29.9% | 34.1% |
|  |  |  |  |  |  |  |  |  |  |
| **Hypertension** | **N** | 199,648 | 267,553 | 292,451 | 327,356 |  | 263,787 | 285,929 | 308,095 |
|  | **n** | 70,559 | 138,464 | 163,362 | 198,267 |  | 134,698 | 156,840 | 179,006 |
|  | **IR (%)** | 35.3% | 51.8% | 55.9% | 60.6% |  | 51.1% | 54.9% | 58.1% |

N: Total number of inspected admissions (denominator); n: Number of admissions with non-recorded condition (numerator); IR (%): Incidence rate (per 100 admissions).

In-between admissions included all admissions between the earliest (first index admission, excluded) and the latest (last index admission, inclusive) admissions with the recorded chronic condition.

Follow-up admissions included any admissions with non-recorded conditions occurring within a restricted follow-up period after the last index admission.

Buffer was the time to the end of study period (30^th^ June 2014). Any follow-up admission occurring in this period was excluded.

* The employed scenario for statistical analysis within this study. Slightly higher numbers of admissions were reported because of inclusion of cases with missing items in covariates.
